# Epigenome-Wide Association Study in Peripheral Tissues Highlights DNA Methylation Profiles Associated with Episodic Memory Performance in Humans

**DOI:** 10.3390/biomedicines10112798

**Published:** 2022-11-03

**Authors:** Yasmine Sommerer, Valerija Dobricic, Marcel Schilling, Olena Ohlei, David Bartrés-Faz, Gabriele Cattaneo, Ilja Demuth, Sandra Düzel, Sören Franzenburg, Janina Fuß, Ulman Lindenberger, Álvaro Pascual-Leone, Sanaz Sedghpour Sabet, Cristina Solé-Padullés, Josep M. Tormos, Valentin Max Vetter, Tanja Wesse, Andre Franke, Christina M. Lill, Lars Bertram

**Affiliations:** 1 Lübeck Interdisciplinary Platform for Genome Analytics (LIGA), University of Lübeck, Ratzeburger Allee 160, 23562 Lübeck, Germany; 2 Department of Medicine, Faculty of Medicine and Health Sciences, Institute of Neurosciences, University of Barcelona, Campus Clínic August Pi i Sunyer, Casanova, 143, 08036 Barcelona, Spain; 3 Institut Guttmann, Institut Universitari de Neurorehabilitació adscrit a la UAB, Garcilaso, 57, 08027 Barcelona, Spain; 4 Departament de Medicina, Universitat Autònoma de Barcelona, Plaça Cívica, Bellaterra, 08193 Barcelona, Spain; 5 Fundació Institut d’Investigació en Ciències de la Salut Germans Trias i Pujol, Camí de les Escoles, Badalona, 08916 Barcelona, Spain; 6 Biology of Aging Working Group, Department of Endocrinology and Metabolic Diseases, Division of Lipid Metabolism, Charité—Universitätsmedizin Berlin (corporate member of Freie Universität Berlin and Humboldt-Universität zu Berlin), Augustenburger Platz 1, 13353 Berlin, Germany; 7 Berlin Institute of Health Center for Regenerative Therapies, Charité—Universitätsmedizin Berlin, Charitéplatz 1, 10117 Berlin, Germany; 8 Center for Lifespan Psychology, Max Planck Institute for Human Development, Lentzeallee 94, 14195 Berlin, Germany; 9 Institute of Clinical Molecular Biology, Christian-Albrechts-University of Kiel, Christian-Albrechts-Platz 4, 24118 Kiel, Germany; 10 Hinda and Arthur Marcus Institute for Aging Research, Hebrew SeniorLife, Harvard Medical School, 1200 Centre St., Boston, MA 02131, USA; 11 Berenson-Allen Center for Noninvasive Brain Stimulation, Beth Israel Deaconess Medical Center, Harvard Medical School, 330 Brookline Ave, Boston, MA 02215, USA; 12 Institute of Epidemiology and Social Medicine, University of Münster, Domagkstr. 3, 48149 Münster, Germany; 13 Ageing Epidemiology Research Unit (AGE), School of Public Health, Imperial College London, Charing Cross Hospital, St Dunstan's Road, London W68RP, UK; 14 Center for Lifespan Changes in Brain and Cognition (LCBC), Department of Psychology, University of Oslo, Forskningsveien 3A, 0373 Oslo, Norway

**Keywords:** DNA methylation, CpG, epigenome-wide association study, EWAS, episodic memory, cross-sectional, longitudinal

## Abstract

The decline in episodic memory (EM) performance is a hallmark of cognitive aging and an early clinical sign in Alzheimer’s disease (AD). In this study, we conducted an epigenome-wide association study (EWAS) using DNA methylation (DNAm) profiles from buccal and blood samples for cross-sectional (*n* = 1019) and longitudinal changes in EM performance (*n* = 626; average follow-up time 5.4 years) collected under the auspices of the Lifebrain consortium project. The mean age of participants with cross-sectional data was 69 ± 11 years (30–90 years), with 50% being females. We identified 21 loci showing suggestive evidence of association (*p* < 1 × 10^−5^) with either or both EM phenotypes. Among these were *SNCA*, *SEPW1* (both cross-sectional EM), *ITPK1* (longitudinal EM), and *APBA2* (both EM traits), which have been linked to AD or Parkinson’s disease (PD) in previous work. While the EM phenotypes were nominally significantly (*p* < 0.05) associated with poly-epigenetic scores (PESs) using EWASs on general cognitive function, none remained significant after correction for multiple testing. Likewise, estimating the degree of “epigenetic age acceleration” did not reveal significant associations with either of the two tested EM phenotypes. In summary, our study highlights several interesting candidate loci in which differential DNAm patterns in peripheral tissue are associated with EM performance in humans.

## 1. Introduction

Episodic memory (EM) is a form of long-term declarative memory, which is involved in the storage and retrieval of unique experiences in life, including information about the location and time of events [1]. It is well established that a decline in EM performance is a hallmark of cognitive aging [2,3,4] and human memory disorders, including amnestic mild cognitive impairment (MCI) [5,6] and Alzheimer’s disease (AD) [5,7]. While several genome-wide association studies (GWASs) have already been performed to evaluate the role of genetic variants in general cognitive function [8,9] and EM performance [10,11,12], the latter highlighting *KIBRA* [10,12] and *CTNNBL1* [11,13] as potential candidate genes, the association between epigenetic modifications in the genome and EM performance still remains largely unknown. DNA methylation (DNAm) is the most commonly studied epigenetic mark in humans, owing to the relative technical ease and high cost-efficiency of measuring DNAm profiles on a genome-wide scale. To this end, several recent studies aimed at the identification of novel biomarkers for cognitive decline [14,15], pre-symptomatic dementia [16], and dementia risk [17] using DNAm profiles derived from blood. In a related line of research, some studies have suggested an association of cognitive performance with accelerated epigenetic aging when comparing DNAm-based age estimates with chronological age [18,19]. Together, this prior work supports the hypothesis that there may be discernible DNAm patterns in the genome associated with cognitive function [20,21,22,23].

In this study, we performed comprehensive epigenome-wide association study (EWAS) analyses on cross-sectional (*n* = 1019) and longitudinal (*n* = 626) changes in EM performance in two datasets assembled under the auspices of the Lifebrain consortium project [24]. Analyzed samples had a mean age of 69 ± 11 years (30–90 years) and an average follow-up period of 5.4 ± 0.5 years for the longitudinal EM phenotype. Genome-wide DNAm profiles were generated from buccal swabs and whole blood using the Infinium MethylationEPIC microarray (featuring ~850 K individual CpG sites) and highlighted 21 CpGs showing at least suggestive (*p* < 1 × 10^−5^) evidence of association with cross-sectional and/or longitudinal changes in EM performance. To our knowledge, our study represents the first EWAS using the EPIC array investigating both cross-sectional and longitudinal changes in EM performance. Our analyses highlight several functionally interesting candidate loci showing altered DNAm patterns with respect to EM performance.

## 2. Materials and Methods

Please see Supplementary Figure S1 for an overview of all analyses that were performed in this study.

### 2.1. Human Samples and Measurements of Episodic Memory

In this study, we analyzed a total of 1019 samples from two independent datasets (Berlin Aging Study II (BASE-II) and Barcelona Brain Health Initiative (BBHI)) with EM and genome-wide methylation data available.

Berlin Aging Study II (BASE-II) and GendAge Study: The BASE-II dataset used in this study consists of older residents (60–85 years of age) from the greater metropolitan area of Berlin, Germany. Cognitive assessments at baseline were performed as part of the BASE-II study [25,26] and follow-up assessments were part of the GendAge study [27]. Overall, at follow-up, there were up to *n* = 800 samples (buccal (*n* = 678) and blood (*n* = 800), both sampled as part of the follow-up assessments) from BASE-II with test results for cross-sectional (buccal (*n* = 678) and blood (*n* = 800), overlap of *n* = 656 samples with data for both tissues) and longitudinal changes in episodic memory performance (buccal (*n* = 626) and blood (*n* = 735), overlap of *n* = 605 samples with data for both tissues) available for DNAm profiling, of which 656 individuals had both buccal and blood samples available (Table 1). The BASE-II/GendAge studies were conducted in accordance with the Declaration of Helsinki and approved by the ethics committee of the Charité—Universitätsmedizin Berlin (approval numbers: EA2/144/16, EA2/029/09) and the Max Planck Institute for Human Development, Berlin (approval number: LIP-2012-04). All participants gave written informed consent before participating. For more information on the EM assessments, see Supplementary Material.

Barcelona Brain Health Initiative (BBHI): The Barcelona Brain Health Initiative (BBHI) is an ongoing, longitudinal study with the focus on evaluating factors determining brain health [28]. Overall, there were 341 buccal samples from BBHI with test results for cross-sectional EM performance available for DNAm profiling. Participants had an age range of 30 to 67 years (Table 1). The BBHI project was conducted in accordance with the Declaration of Helsinki and following the recommendations of the “Unió Catalana d’Hospitals” with written informed consent from all subjects. The protocol was approved by the Unió Catalana d’Hospitals (approval number: CEIC 17/06). For more information on the EM assessments, see Supplementary Material.

### 2.2. Episodic Memory Phenotypes

For the cross-sectional EM phenotype, the first principal component (PC) of memory test performances (see above and Supplementary Material) was calculated with a principal component analysis (PCA) using the PCA function in the R package FactoMineR (version 2.6) [29]. This variable (PC1) was corrected for age at the time of the assessment used for the cross-sectional phenotype using a linear regression model performed with the lm function in R. The residuals of this regression were used as outcome phenotypes in the cross-sectional EM EWAS analyses. For the longitudinal change in the EM phenotype, an annual percentage change (APC) estimate was calculated for each memory test. As expected, we observed a decrease in the test performance over time (average interval: 5.4 years) between baseline measurement and follow-up in the BASE-II dataset (Supplementary Figure S2). See Supplementary Material for more details.

### 2.3. DNA Extraction and Processing

Genomic DNA was extracted using commercial kits for blood (Plus XL manual kit, LGC, London, UK), or buccal samples (Buccal-Prep Plus DNA Isolation Kit, Isohelix, Harrietsham, UK) following the manufacturer’s instructions. To assess the concentration and purity of the obtained DNA, we used a NanoDrop ONE spectrophotometer (Thermo Fisher Scientific, Waltham, MA, USA). See Supplementary Material for more details.

### 2.4. DNA Methylation Profiling

DNAm profiling was performed at IKMB at UKSH campus Kiel using the “Infinium MethylationEPIC” array (Illumina, Inc., San Diego, CA, USA), as described previously [23]. After calling the raw DNAm intensities with the iScan control software (version 2.3.0.0; Illumina, Inc., San Diego, CA, USA), these data were exported in idat format for downstream processing and analysis. See Supplementary Material for more details.

### 2.5. DNA Methylation Data Processing and Quality Control

DNAm data processing and quality control (QC) were performed using the same procedures as described previously [23] unless noted otherwise using the R (version 3.6.1) package bigmelon (version 1.22.0) with default settings [30]. Cell-type composition estimates were obtained with the R package EpiDISH (version 2.12.0) [31], followed by correction of the DNAm values for cell-type composition with the removeBatchEffect function in the R package limma (version 3.52.4) [32]. For all statistical analyses, the DNAm β-values were used. See Supplementary Material for more details.

### 2.6. Epigenome-Wide Association Analyses to Identify Differentially Methylated Probes

Statistical analyses to identify differentially methylated probes (DMPs) were performed in each dataset separately using linear regression models performed by the lm function in R and the EM phenotype (residuals of the first PC regressed on age, see above) as a continuous outcome variable. To account for differences in the DNAm profiles due to technical (e.g., laboratory batch, microarray) and other factors, we performed a PCA on a subset of uncorrelated CpGs in the cell-type corrected data as described previously [23] and included these DNAm PCs as covariates in the regression model. See Supplementary Material for more details.

### 2.7. Calculation of Poly-Epigenetic Scores (PESs) for General Cognitive Abilities and AD

PESs were calculated for each individual based on the test statistics from a published blood-based EWAS on cognitive abilities [15], on all phenotypes that were evaluated in that publication: general cognitive ability (g), general fluid cognitive ability (gf), vocabulary, digit symbol test score (digit), logical memory (LM), and verbal fluency (verbal). Linear regression models were fitted to the cross-sectional and longitudinal changes in EM on PESs, adjusting for the same covariates as in the primary EWAS. See Supplementary Material for more details.

### 2.8. Epigenetic Age Estimation

To estimate the “epigenetic age” (i.e., DNAm age), we applied the “Horvath multi-tissue predictor” (HMTP) using the R script provided in the primary publication [33]. DNAm age acceleration is defined as the residual of a linear regression of epigenetic age on chronological age. This estimate of epigenetic age acceleration in our study was used as an independent variable to predict EM performance using the same linear models as for the primary EWAS analyses. Cell-type composition estimates according to the R-package EpiDISH [31] were included as a covariate in the linear regression analysis. See Supplementary Material for more details.

### 2.9. Look-Up of EM-Associated CpGs in an Independent EWAS on AD-Related Phenotypes in Human EC

To further characterize the CpGs with suggestive evidence of association with EM, we used test statistics from our recent AD EWAS of DNAm in the EC [23]. Results for EM-associated CpGs identified in this study were obtained for the Braak stage and AD case–control analyses generated in our previous study [23]. Additional association results were retrieved from EWASs on the AD Braak stage [34,35,36,37] and cognition [15]. See Supplementary Material for more details.

### 2.10. Look-Up of EM-Associated CpGs in an Independent Buccal–Brain Correlation Map

To estimate whether the DNAm patterns observed in this study could potentially also be seen in the primary tissue of interest for cognition—the brain—we used data from the correlation map of DNAm profiles ascertained from 120 matched human brain and buccal samples recently generated by our group [38]. Samples used in these buccal–brain DNAm correlation analyses are independent from those use here.

### 2.11. Look-Up of EM-Associated CpGs in a Buccal mQTL Database

To investigate the potential genetic influence on the DNAm at the EM-associated CpGs, we used an in-house buccal mQTL database, which is based on 837 buccal samples from the BASE-II dataset, out of which 675 samples were also used in this EWAS (manuscript in preparation). Briefly, for the buccal dataset, both genome-wide QC’ed DNAm profiles (761,034 CpG-sites) and SNP genotyping data (7,663,257 SNPs) were available for mQTL analysis. For cis mQTL computations (with cis mQTLs defined as within ±1 Mb of the CpG site), we used the matrix eQTL software (version 2.3) [39], which performed an additive linear model with sex, genetic PCs 1 to 5, DNAm PCs 1 to 10, and genotyping batch as covariates. Before association analysis, genome-wide DNAm profiles were adjusted for cell type composition estimates. Cis mQTLs with *p* < 1.00 × 10^−15^ were defined as genome-wide-significant for this arm of our analyses.

### 2.12. DNAm–mRNA Correlation Analyses

To estimate whether the DNAm patterns of CpGs showing suggestive association with EM in this study correlated with gene expression in human brain samples, we correlated DNAm status with RNA sequencing results generated in EC samples from healthy controls from ref. [23]. The analyses performed here entailed computing Spearman rank correlations using R’s cor.test function between DNAm of a CpG and normalized RNA-seq data of the respective annotated gene(s), with annotations to specific gene regions according to the Illumina manifest (version 1.0 B5) for the EPIC array and the GREAT annotation tool (v4.0.4) [40]. Multiple testing was accounted for by computing thresholds using the false-discovery rate (FDR) applying the Benjamini–Hochberg method. See Supplementary Material for more details.

## 3. Results

### 3.1. EM Performance Measures in the Analyzed Datasets

For the cross-sectional EM performance phenotype, we had data from 1019 participants available for the meta-analysis across datasets from two study centers, with a mean age of 69 years ± 11 years. The longitudinal change in EM performance was available for 626 buccal and 735 nonoverlapping blood samples in the BASE-II dataset, with a mean age at baseline of 70 ± 4 years and an average of 5.4 ± 0.5 years between baseline and follow-up assessments. For an overview of the samples available for the cross-sectional and longitudinal EM EWAS analyses, see Table 1. Overall, the cross-sectional EM performance and longitudinal change in EM performance correlated moderately (*R* = 0.42, *p* = 1.87 × 10^−35^). For a visual summary of the longitudinal change in EM performance in the BASE-II dataset, see Supplementary Figure S2. As expected, there was a tendency toward lower test scores at the follow-up timepoint when compared to baseline analyses for all four tests. A paired t-test on the change in EM performance over time revealed that test scores differed significantly between both time points for three out of four tests applied in this dataset (see legend to Supplementary Figure S2).

### 3.2. EWAS Meta-Analyses Highlight Several CpG Loci Showing at Least Genome-Wide Suggestive Association with Cross-Sectional and Longitudinal Changes in EM Performance

**Figure 1:**
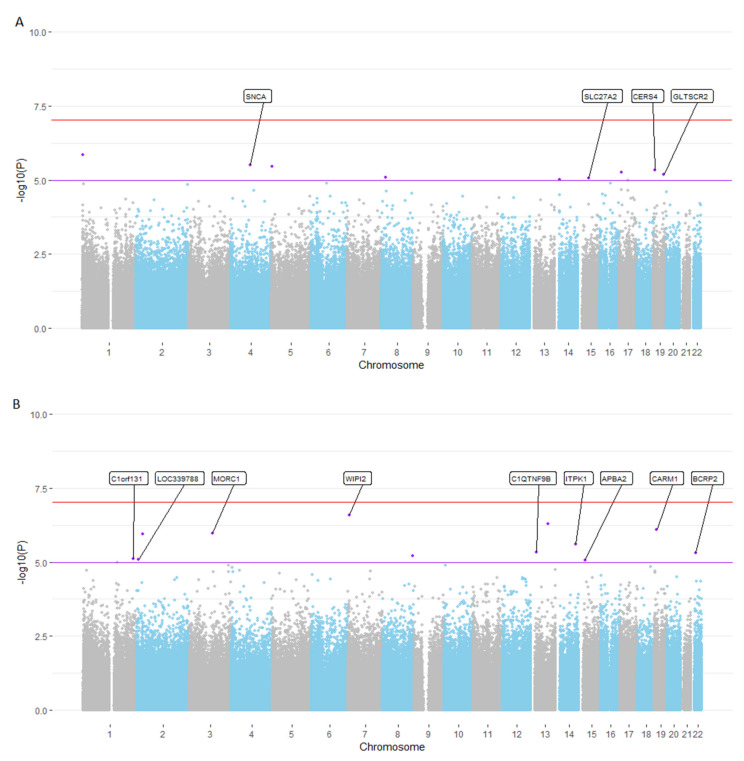
Manhattan plot for EWAS meta-analysis using cross-sectional (**A**) and longitudinal change in (**B**) EM performance. Figure 1 legend: Panel (**A**): Cross-sectional EM performance EWAS; panel (**B**): Longitudinal change in EM performance EWAS; the red line indicates the epigenome-wide significance threshold of 9 × 10^−8^ according to Mansell et al. [41], whereas the purple line indicates the suggestive significance threshold of 1 × 10^−5^. CpGs with suggestive evidence of association are marked in purple and annotated with the gene name according to the Illumina manifest (version 1.0 B5). CpGs without gene name were not annotated to a gene in this manifest. Chromosome = number of human chromosome without sex chromosomes.

Overall, our EWAS analyses displayed low genomic inflation factors λ, with a maximum λ of 1.03 (Supplementary Table S1). While the EWAS analyses performed in this study did not identify any epigenome-wide significant signals (*p* < 9 × 10^−8^; according to Mansell et al. [41]; Figure 1), they highlighted several loci showing suggestive (*p* < 1 × 10^−5^) evidence of association with cross-sectional or longitudinal EM performance. Specifically, in the EWAS of cross-sectional EM performance, we identified nine loci showing evidence at this level (Figure 1A). Three of these loci also showed at least nominally significant association (*p* < 0.05) with a longitudinal change in EM performance (Table 2A), with effects pointing in the same direction in all instances. For the longitudinal change in EM performance, we observed a total of twelve suggestive EWAS signals (Figure 1B; Table 2B), six of which also showed at least nominally significant association (*p* < 0.05) with cross-sectional EM performance with the same direction of effect (Table 2B).

Notably, our EWAS on cross-sectional and longitudinal changes in EM performance highlighted four genes, *IRX2*, *SEPW1*, *HDC*, and *ITPK1,* that were previously described in the context of other independent EWASs utilizing the AD case–control status and Braak stage as outcomes [34,42,43]. Other genes highlighted by this study include *CD320*, which was reported to be associated with Huntington’s disease in a previous EWAS [44]; *USP43*, which was shown to have DNAm levels associated with progressive supranuclear palsy [45]; *APBA2*, which was reported in an EWAS of Parkinson’s disease (PD) [46] (Supplementary Table S2). In addition, cg17268483 (annotated with the genes *SLC27A2* and HDC) showed association at a nominal *p*-value (*p* < 0.05) with an AD Braak stage and case–control status in our previous AD EWAS in the EC [23] (Supplementary Table S2). Lastly, our look-up of the EWAS results on cognitive performance recently reported by McCartney et al. [15] showed that three CpGs (cg18370700 [*IRX4/IRX2*], cg17268483 [*SLC27A2/HDC*], and cg19857541 [*MORC1*]) were also at least nominally significantly associated with at least one phenotype in the McCartney et al. study displaying the same direction of effect (Supplementary Table S2). Taken together, there is a high level of concordance between previous EWAS results on EM and traits related to neurodegenerative diseases at 1/3 (i.e., 7 of 21) of the EM EWAS loci highlighted here, supporting the overall plausibility (and perhaps relevance) of our results. In addition, two of the EM EWAS loci, cg12160320 and cg13468767, also showed a moderate to high correlation of DNAm between matched prefrontal cortex and buccal samples according to our recently derived buccal–brain correlation map [38] (Supplementary Table S2).

Finally, we determined the overlap between suggestive EWAS results generated here and DNAm loci associated with SNP genotypes from a methylation quantitative trait locus (mQTL) database comprising results on 837 individuals recently generated in buccal samples by our group (data not shown; m.s. in preparation). This look-up revealed that DNAm at the majority (17/21, 81%) of CpGs suggestively associated with EM was under genetic control at *FDR* = 0.05, i.e., all CpGs except cg08891989, cg16525470, cg05275832, and cg19100344 (rows highlighted in grey in Supplementary Table S2). Thus, these four nongenetic CpGs may be regulated predominately by nongenetic factors.

### 3.3. Poly-Epigenetic Score Analysis for Cognitive Abilities and AD Show Only Little Correspondence with Episodic Memory EWAS

The number of CpGs used for the calculation of each phenotype PES and *p*-value threshold can be found in Supplementary Table S3. To assess how well our novel EM-based EWAS results compare to EWASs from other efforts beyond looking up individual CpG results, we calculated PESs based on summary test statistics from a recently completed blood-based EWAS on cognitive abilities [15], and our own AD EWAS in the human brain (EC) [23]. These analyses served the purpose of assessing the potential overlap in epigenetic signatures across different but related phenotypes, e.g., cognitive performance in domains other than EM and the presence of AD. Each of these PESs were then tested for association with cross-sectional and longitudinal changes in EM performance in our datasets (Supplementary Tables S4 and S5, respectively).

Overall, we observed only a very modest concordance between these prior studies and our current EM results. Upon comparing the results from McCartney et al. [15] to the cross-sectional EM performance (Supplementary Table S4), we found that only up to 0.8% of variance in cross-sectional EM performance could be explained by the blood-based PES on general cognitive function (*p* = 0.01), and up to 0.6% of variance in cross-sectional EM performance could be explained by the blood-based PES on the vocabulary phenotype (*p* = 0.03). None of these associations remained significant after adjustment for multiple testing using Bonferroni correction for ten independent tests (*p* = 0.12 and *p* = 0.34, respectively). Furthermore, the buccal-based PES did not show any association with cross-sectional EM performance. This is not unexpected, considering that the test statistics used for the cognition PES calculations were originally generated in blood-based DNAm profiles of McCartney et al. [15]. Likewise, there were no clearly discernible signals upon testing the cognition PES with longitudinal changes in EM performance in our dataset using both blood and buccal specimens (Supplementary Table S5). Again, this is not unexpected, given that the test statistics used for the PES calculations are based on cross-sectional (and not longitudinal) cognition phenotypes of McCartney et al. [15].

Lastly, for the AD EWAS-derived PES, we found several associations of the buccal-based scores with cross-sectional EM performance, but not a longitudinal change in EM performance (Supplementary Table S4). In contrast to the cognition PES described in the previous paragraph, these associations remained significant after adjustment for multiple testing (9.75 × 10^−3^ < *p*_adj_ < 0.01, with up to 2.8% of the variance in cross-sectional EM performance explained by the AD PES). In summary, none of the performed PES analyses point toward a large overlap in associated DNAm patterns across the EM phenotypes considered here. The best concordance in this context was achieved by the buccal-based AD PES and cross-sectional EM performance.

### 3.4. Correspondence of EWAS Results in Blood and Buccal Samples Underscores Tissue Specificity

For the BASE-II dataset, both buccal and blood samples were available, which allowed for a detailed evaluation of the tissue-specificity of the DNAm patterns associated with EM performance of our study. To this end, we compared the buccal-based EWAS results generated in this dataset (cross-sectional *n* = 678; longitudinal *n* = 626) with those obtained from EWASs in blood from a largely overlapping set of individuals (cross-sectional *n* = 800; longitudinal *n* = 735). Correlating the BASE-II EWAS test statistics from the buccal samples (with the two batches of buccal samples meta-analyzed) and the blood samples only showed modest correlation coefficients (cross-sectional: *r* = 0.05, *p* < 2.23 × 10^−308^; longitudinal: *r* = 0.03, *p* = 1.6 × 10^−197^), arguing that the two different peripheral tissue types (blood and buccal) derived from the same individuals at the same timepoint capture different aspects of DNAm variance related to brain function.

### 3.5. Correlation Analysis between DNAm Levels and mRNA Expression in Human Brain Samples Highlights Three Loci

To assess whether gene expression in the brain correlates with DNAm at the CpG sites suggestively associated with EM performance in the meta-analysis for buccal samples, i.e., whether DNAm at these CpGs is associated with the gene expression of annotated genes, we used a dataset of matched RNA-seq and DNAm profiles from the EC, and calculated Spearman rank correlations for CpG-gene pairs as described previously [23]. While CpGs were selected from the primary EWAS results in buccal samples, the correlation with mRNA levels in the brain was calculated using DNAm data generated in the same brain samples. This resulted in the evaluation of 23 CpG-gene pairs (Supplementary Table S6), of which two showed significant Spearman rank correlations at *FDR* = 0.05. According to this analysis, DNAm at cg27184903 was positively correlated with the expression of APBA2 (*ρ* = 0.34, *q* = 4.7 × 10^−4^) and NDNL2 (*ρ* = 0.34, *q* = 4.7 × 10^−4^). While the number and strength of the associations identified in this analysis were overall modest, we note that they were generated in an entirely independent dataset and may still provide some initial functional clues with respect to the EM EWAS signals observed in the main analyses of our study.

### 3.6. Horvath Epigenetic Age Acceleration Is Not Associated with EM Performance

**Figure 2:**
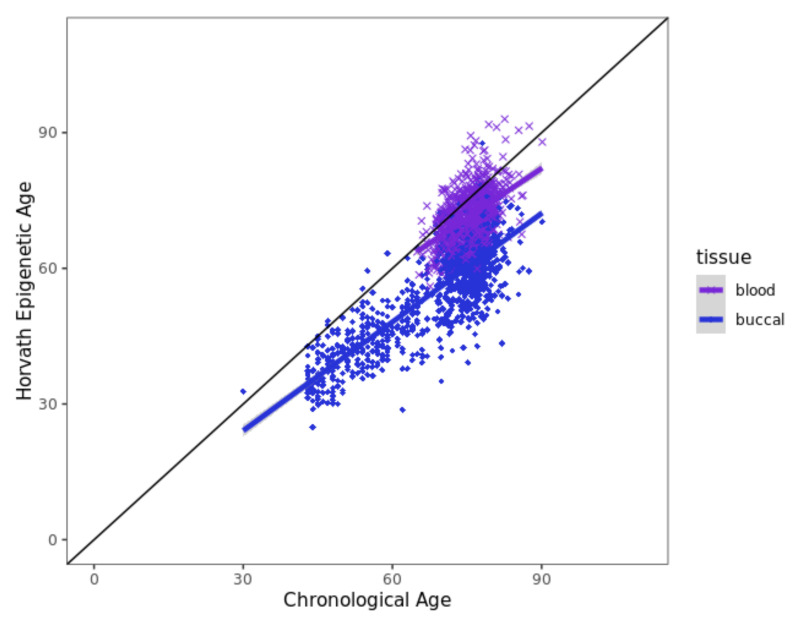
Scatterplot of chronological age and HMTP DNAm age estimates. Figure 2 legend: Samples were colored according to tissue (dark blue: buccal; purple: blood); the black line denotes perfect concordance between chronological age (years) and Horvath epigenetic age (years).

To probe for a potential correlation between DNAm age acceleration and EM performance in our datasets, we estimated DNAm age in our dataset using the HMTP “clock”. As expected and reported previously [33,47], the HMTP DNAm age estimates were highly correlated with chronological age for all datasets included in our study (Figure 2, Pearson correlation coefficients r: 0.29–0.50, 2.56 × 10^−51^ < *p* < 8.77 × 10^−9^; Supplementary Table S7A). Interestingly, comparing these DNAm age estimates in the 656 BASE-II individuals having both blood and buccal-derived DNAm data available revealed that the correlation of chronological age and DNAm age was considerably higher in the blood dataset (*r* = 0.49, *p* = 2.49 × 10^−41^, Supplementary Figure S3A) when compared to the buccal-based estimates (*r* = 0.31, *p* = 3.14 × 10^−16^, Supplementary Figure S3B). These results indicate that the HMTP clock performs better in blood-based DNAm samples, while it tends to underestimate DNAm ages with respect to chronological age in other tissues, particularly buccal-derived DNA, which is in line with previously reported results [33,48]. Notwithstanding this “technical verification” of the DNAm age estimation approach, none of the analyzed datasets showed a noteworthy association between DNAm age acceleration and cross-sectional or longitudinal changes in EM performance (Supplementary Table S7B, Supplementary Figure S4). This means that DNAm age acceleration does not appear to be a useful biomarker for either cross-sectional or longitudinal changes in EM performance, at least not in the datasets analyzed here and when employing the Horvath clock algorithm.

## 4. Discussion

In this study, we performed a number of comprehensive EWAS analyses probing for potential associations between genome-wide DNAm profiles and cross-sectional and longitudinal changes in EM performance in two carefully phenotyped datasets of the Lifebrain consortium. While we identified no signals passing a recently proposed epigenome-wide significance threshold for the EPIC array [41], we identified several loci showing at least suggestive evidence of association (*p* < 1 × 10^−5^) with either or both EM phenotypes. Intriguingly, several of our suggestive EM EWAS signals were previously reported to be associated with cognitive functioning or neurodegenerative diseases, such as AD, Huntington’s disease, or PD, in EWAS and GWAS (Supplementary Table S1). In particular, this relates to the CpGs located close to the genes *SNCA*, *SEPW1*, *ITPK1*, and *APBA2*, which we consider the functionally most interesting findings of our study and which we briefly discuss in the subsequent section.

*SNCA* encodes the neuronal protein “alpha-synuclein”, which is involved in the synaptic activity of neurons by regulating the release of neurotransmitters [49,50,51,52]. Aberrant polymerization of the protein is a hallmark of several neurodegenerative diseases, including PD and Lewy Body Dementia [53]. Genetic variants in *SNCA* are established risk factors for PD (e.g., ref. [54,55]) and Lewy Body Dementia (e.g., ref. [56]). In addition, recent work suggests that transgenic mice with the A53T mutation in *SNCA* displayed learning and memory deficits, providing a potential direct link between *SNCA* and cognitive function [57]. *SEPW1* encodes “selenoprotein W”, which has been shown to be expressed in the brain, among other tissues [58]. It is involved in protecting cells from oxidative stress during the development of the nervous system. Furthermore, previous EWASs reported associations of CpGs annotated to *SEPW1* with Huntington’s disease [44] and AD [34]. Knockout of the protein in mice resulted in changes in the amygdala and hippocampus, as well as impaired fear memory [59]. *ITPK1*, which encodes “inositol-tetrakisphosphate 1-kinase”, is an enzyme that belongs to the inositol 1,3,4-trisphosphate 5/6-kinase family [60]. Higher phosphorylated forms of inositol in mammalian cells are essential for life, and animal models show that reduced *ITPK1* levels can cause neural tube defects [61]. A recent proteomics study in human brain samples from the ROS/MAP dataset showed an association between levels of *ITPK1* protein and cognitive decline [62]. This fits to DNAm data from a largely overlapping set of individuals, i.e., the EWAS by Zhang et al., also showing a highly significant association between methylation patterns near *ITPK1* and Braak staging [34]. In follow-up work, the ROS/MAP group later explained the association between *ITPK1* and cognitive decline to be mediated by microstructural changes in the brain [63]. Lastly, *ABPA2* encodes for “amyloid beta precursor protein binding family A member 2”, which was reported to play a crucial role in synaptic vesicle exocytosis [64]. In addition, it binds to the amyloid precursor protein (APP), a hallmark protein in AD pathogenesis, modulating its proteolytic fragmentation [65]. Overexpression of *ABPA2* has been shown to lead to a decrease in Aβ secretion in mice [66], along with protecting them from and rescuing memory deficits in an AD mouse model [67]. In addition to its ties to AD, differential DNAm at the *ABPA2* locus was reported to be associated with PD [46].

The strengths of our study lie in the availability of both cross-sectional and longitudinal changes in EM performance data, the combination of two independent and carefully phenotyped EWAS datasets, leading to comparably large overall sample sizes (cross-sectional *n* = 1019; longitudinal *n* = 626), and the application of stringent quality control and data processing procedures (as evidenced by relatively low genomic inflation factors λ for all our analyses; Supplementary Table S1) minimizing the risk for spurious association signals. Furthermore, in addition to performing the primary EWAS analyses using EM as an outcome, we also performed a range of auxiliary analyses with the goal to facilitate the interpretation of our main findings. This included comparing our results with a recent blood-based EWAS on cognitive abilities [15] by calculating PESs. While in the buccal datasets, these only showed limited concordance with our EM phenotypes, this correlation improved when using the blood-based DNAm data. However, we note that this improvement was only minor, suggesting that other factors (such as phenotype definition or technical aspects) may have contributed to the differences in EWAS results. Second, while we generally observed high correlations between Horvath multi-tissue DNAm age and estimates and chronological age in all datasets, we found no significant association of epigenetic age acceleration with either cross-sectional or longitudinal changes in EM performance. While this finding is in agreement with some previous data [20,68], it does not agree with the results from Marioni et al. [18] and Zheng et al. [19], which are similar to our study in many aspects. For instance, the authors of ref. [18] also used HMTP age acceleration, cross-sectional (but not longitudinal) cognitive performance, and physical fitness and analyzed a dataset with a similar age range (70–76 years) and sample size (cross-sectional *n* = 920) generated from blood-based DNAm profiles. They found a significant association between DNAm age acceleration and fluid-type general intelligence (*p* = 0.024). Given the low concordance between PES-based analyses constructed from data in ref. [15] and our DNAm profiles (see above), the difference in DNAm clock results, again, possibly relates to differences in the cognitive phenotypes used and/or technical differences across studies. Lastly, we correlated DNAm and RNA sequencing data recently generated by our group in an independent dataset of EC-based brain samples [23]. While, overall, this only revealed a modest degree of correlation across all CpG-mRNA pairs analyzed (Supplementary Table S2), one locus (cg27184903) in particular showed a significant correlation between DNAm and gene expression levels of *APBA2*. Specifically, the results of these analyses suggest that increases in DNAm at CpG cg27184903 lead to an increase in *APBA2* mRNA expression, providing a direct potential mechanistic link of this finding to EM performance.

Possible limitations of our study include the following aspects. First, all DNAm profiles are based on “bulk” tissue, making it difficult to decipher whether and which of the observed association signals are dependent on changes in cell type composition. To address this issue, we estimated the cell type composition using current reference databases for blood and epithelial tissues [31] and corrected our DNAm data directly for these estimates. EWAS results only changed marginally, so it is unlikely that inter-individual variations in cell-type composition have substantially affected our results. We also note that cell counts determined directly in the laboratory (available for the blood samples of the BASE-II dataset) correlated very highly with equivalent cell-type composition estimates derived in silico from the DNAm data (Supplementary Figure S5), underscoring the validity of the deconvolution methods and reference datasets used here. However, future studies using single-cell-based DNAm profiling would be needed to more extensively address this problem. Second, as with all EWASs, we are unable to disentangle cause–effect relationships related to our results, i.e., the observed changes in DNAm patterns could either represent the consequence or cause for the observed changes in EM performance. However, as one of the overarching aims of our study was to probe for novel (epigenetic) biomarkers of cognitive performance in easily accessible, nonbrain tissues, causality relationships are not a major concern. Third, while we went to great lengths to control for confounding factors due to unknown factors in our study, we cannot exclude the possibility that some signals reported here reflect spurious associations due to residual (and uncontrolled) confounding factors. However, given the overall very low degree of inflation of the epigenome-wide test statistics across essentially all our analyses, we do not expect such confounding factors to have affected our results to a large extent. Finally, we only observed limited overlap of our EWAS signals with previous EWASs on cognitive abilities, in particular, those from ref. [14] and ref. [15]. This might be due to the fact that previous EWASs did not specifically use EM as a cognitive trait (but rather tested other domains of cognitive functioning) and that all previous EWASs in the field utilized DNAm profiles generated from blood, but not buccal samples. With respect to the latter point, we note that the PES analyses using blood-based EWAS results for cognitive functioning from ref. [15] also only showed a very modest overlap with our blood-derived DNAm data (Supplementary Table S4). This observation argues for genuine differences in DNAm patterns between our EM phenotypes and the cognitive phenotypes utilized in ref. [15], which are not due to the use of a different peripheral tissue.

In conclusion, our EWAS on cross-sectional and longitudinal changes in EM performance highlighted several loci with at least suggestive evidence of association. These include *SNCA*, *SEPW1*, *ITPK1*, and *APBA2*, which have all been previously implicated to relate to neurodegenerative diseases and/or cognitive functioning. Future studies are needed to validate our findings, to further elucidate their potential implications in brain function, and to assess the utility of the highlighted CpGs as potential molecular biomarkers of cognitive performance.

## Figures and Tables

**Table 1:** Summary statistics and description of the samples used in the cross-sectional (A) and longitudinal changes in (B) EM performance analyses.

A: Cross-sectional EM performance.						
Analysis	*N*	% F	Age (mean ± sd)	Age range	DNAm PCs	Genetic PCs
BASE-II buccal-1	433	52	76 ± 4	65–86	12	11
BASE-II buccal-2	245	50	76 ± 4	66–90	9	11
BBHI	341	46	54 ± 7	30–67	11	4
Meta-analysis	1019	50	69 ± 11	30–90	NA	NA
BASE-II blood	800	50	76 ± 4	65–90	10	13
**B: Longitudinal EM performance.**						
**Analysis**	***N***	**% F**	**Age (mean ± sd)**	**Age range**	**DNAm PCs**	**Genetic PCs**
BASE-II buccal-1	403	52	70 ± 4	61–81	12	11
BASE-II buccal-2	223	50	70 ± 4	61–85	8	7
Meta-analysis	626	51	70 ± 4	61–85	NA	NA
BASE-II blood	735	50	70 ± 4	61–85	9	8

Table 1 legend: %F: percentage of females in the dataset; sd: standard deviation; DNAm: DNA methylation; PCs: principal components; “buccal-1” and “buccal-2” refer to two separate laboratory batches in which the BASE-II samples were analyzed. Samples for BASE-II were obtained during follow-up assessments, with 656 and 605 samples overlapping between buccal and blood samples in the cross-sectional and longitudinal analyses, respectively. Provided ages for BASE-II at follow-up (cross-sectional) and baseline (longitudinal). Meta-analyses are based on buccal samples, and blood samples were used for a separate EWAS.

**Table 2:** Suggestively significant signals (*p* < 1 × 10^−5^) of EWAS meta-analysis with cross-sectional (A) and longitudinal change in (B) EM performance.

A: Cross-sectional EM performance.						
CpG	Location	Gene annotation *	Effect_cross-sect._	p_cross-sect._	Effect_longitudinal_	p_longitudinal_
cg25311963	chr1:1546691	*MIB2*	−7.15	1.41 × 10^−6^	−0.10	0.93
cg15402943	chr4:90659260	*SNCA, TIGD2*	−6.38	3.07 × 10^−6^	−0.81	0.45
cg18370700	chr5:2212396	*IRX4, IRX2*	5.38	3.48 × 10^−6^	0.98	0.28
**cg12160320**	**chr19:8279608**	***CERS4, CD320***	**−5.76**	**4.74 × 10^−6^**	**−2.04**	**0.04**
cg13468767	chr17:9672024	*DHRS7C, USP43*	−2.69	5.60 × 10^−6^	−0.53	0.27
cg27110655	chr19:48259098	*GLTSCR2, SEPW1*	6.90	6.39 × 10^−6^	1.39	0.26
**cg14408927**	**chr8:18875728**	***PSD3***	**4.57**	**7.96 × 10^−6^**	**1.90**	**0.02**
**cg17268483**	**chr15:50477521**	***SLC27A2, HDC***	**13.02**	**8.48 × 10^−6^**	**5.07**	**0.03**
cg02064414	chr14:23122560	*OXA1L, OR6J1*	4.24	9.69 × 10^−6^	0.97	0.21
**B: Longitudinal EM performance.**						
**CpG**	**Location**	Gene annotation*	**Effect_longitudinal_**	**p_longitudinal_**	**Effect_cross-sect._**	**p_cross-sect._**
**cg08891989**	**chr7:5272846**	***WIPI2, SLC29A4***	**8.96**	**2.56 × 10^−7^**	**5.19**	**0.02**
ch.13.1159947F	chr13:78677888	*EDNRB, POU4F1*	−16.43	5.05 × 10^−7^	−5.90	0.16
**cg16525470**	**chr19:10983625**	***CARM1, YIPF2***	**−5.50**	**7.75 × 10^−7^**	**−5.03**	**7.22 × 10^−4^**
**cg19857541**	**chr3:108836800**	***MORC1***	**9.13**	**1.05 × 10^−6^**	**5.93**	**0.01**
**cg05275832**	**chr2:27984686**	***SUPT7L, MRPL33***	**−4.87**	**1.11 × 10^−6^**	**−2.89**	**0.03**
cg14744604	chr14:93559541	*ITPK1, CHGA*	−5.50	2.50 × 10^−6^	−1.36	0.36
**cg19531475**	**chr13:24472454**	***C1QTNF9B***	**−7.04**	**4.55 × 10^−6^**	**−4.28**	**0.02**
cg21390166	chr22:21455845	*BCRP2, SLC7A4, GGT2*	−4.13	4.76 × 10^−6^	−1.84	0.10
cg18632612	chr8:140116347	*COL22A1, KCNK9*	3.70	6.21 × 10^−6^	1.24	0.25
cg19100344	chr1:231372247	*C1orf131, GNPAT*	4.00	7.50 × 10^−6^	1.48	0.21
cg16655166	chr2:8063199	*LOC339788, ID2*	5.04	7.96 × 10^−6^	−0.08	0.96
**cg27184903**	**chr15:29285727**	***APBA2, NDNL2***	**6.01**	**8.79 × 10^−6^**	**6.22**	**3.93 × 10^−4^**

Table 2 legend: Suggestive CpGs (*p* < 1 × 10^−5^) of the EWAS meta-analyses; panel A: cross-sectional EM performance EWAS meta-analysis; bolded entries show association at a nominal level (*p* < 0.05) with longitudinal change in EM; panel B: longitudinal change in EM performance EWAS meta-analysis; bolded entries show association at a nominal level (*p* < 0.05) with cross-sectional EM. * Gene annotation according to the Illumina manifest (v1.0 B5) for the EPIC array and the GREAT annotation tool [40]; CpGs / genes in grey highlight loci with independent prior EWAS evidence (Supplementary Table S1).

## Data Availability

Restrictions apply to the availability of these data. Raw data were generated as part of the GendAge/BASE-II and BBHI studies and are available from the authors upon request and after approval from the GendAge/BASE-II and BBHI steering committees. Summary statistics of the main EWAS meta-analysis results can be accessed from the website of the Lübeck Interdisciplinary Platform for Genome Analytics (LIGA) at URL: https://www.liga.uni-luebeck.de/sommerer_episodic_memory_EWAS_results/.

## Supplementary Materials

The following supporting information can be downloaded at: https://www.mdpi.com/article/10.3390/biomedicines10112798/s1, Figure S1: Trajectories of a random set of 100 individuals for test performance scores at TP0 and TP1 and boxplots of the test performance scores in all BASE-II individuals at baseline (TP0) and follow-up (TP1). Figure S2: Scatterplot of chronological age and HMTP DNAm age estimates for the BASE-II blood dataset (A, *R* = 0.49) and the BASE-II buccal datasets (B, *R* = 0.31) using individuals where both blood and buccal samples (*n* = 656) were available. Figure S3: HMTP age acceleration and cross-sectional (left) and longitudinal changes in (right) EM performance. A-B: BASE-II blood; C-D: BASE-II buccal-1; E-F: BASE-II buccal-2; G: BBHI. Figure S4: Comparison of cell-type compositions for eosinophiles (A), lymphocytes (B), monocytes (C), and neutrophiles (D) reported by a laboratory and estimated by the R-package EpiDISH [31] for the BASE-II blood samples. Figure S5: Comparison of cell-type compositions for eosinophils, lymphocytes, monocytes, and neutrophils reported by a laboratory and estimated by the R-package EpiDISH for the BASE-II blood samples. Table S1: Genomic inflation factors lambda for all EWAS analyses. Table S2: Comparison of CpGs showing suggestive evidence of association with episodic memory performance in independent datasets. Table S3: Number of CpGs used for the cognition PES calculation based on each phenotype and *p*-value threshold. Table S4: Variance in cross-sectional episodic memory performance explained by each cognition PES. Table S5: Variance in longitudinal episodic memory performance explained by each cognition PES. Table S6: Spearman rank correlation of DNAm and gene expression in entorhinal cortex using only healthy controls across *N* = 93 samples. Table S7: A: Pearson correlation of chronological age and Horvath multi-tissue DNAm age; B: test statistics of association tests between DNAm age acceleration and cross-sectional/longitudinal
