# Supplementary material for: Epigenome-Wide Association Study in Peripheral Tissues Highlights DNA Methylation Profiles Associated with Episodic Memory Performance in Humans
Source: Biomedicines. 2022 Nov 3;10(11):2798. doi: 10.3390/biomedicines10112798 (PMC9687249; doi:10.3390/biomedicines10112798)
Supplement: Supplementary file 1 [file biomedicines-10-02798-s001.zip › sommerer_et_al_episodicmemory_EWAS_supplement.revised_by_author.pdf]

Supplementary Material for manuscript:

Epigenome-wide association study in peripheral tissues highlights DNA methylation profiles associated with episodic memory performance in humans

Yasmine Sommerer, Valerija Dobricic, Marcel Schilling, Olena Ohlei, David Bartrés-Faz, Gabriele Cattaneo, Ilja Demuth, Sandra Düzel, Sören Franzenburg, Janina Fuß, Ulman Lindenberger , Álvaro Pascual-Leone, Sanaz Sedghpour Sabet, Cristina Solé-Padullés, Josep M. Tormos, Valentin Max Vetter, Tanja Wesse, Andre Franke, Christina M. Lill and Lars Bertram

Supplementary Methods

Human samples and measurements of episodic memory

Berlin Aging Study II (BASE-II) and GendAge Study: The BASE-II dataset used in this study consists of older residents (60-85 years of age) from the greater metropolitan area of Berlin, Germany. Cognitive assessments at baseline were performed as part of the BASE-II study [1,2] and follow-up assessments were part of the GendAge study [3]. EM performance in each participant was evaluated with four tests: 1. The Verbal Learning and Memory Test (VLMT) [4] assesses auditory verbal learning of 15 words including five learning trials, early recall, an interference list after five learning trials, free recall tests directly after an interference list as well as 30 min later (late recall). The sum of items recalled across trials 1 to 5 provides a measure of overall learning performance which was used here. 2. The Face–Profession Task [5] assesses associative binding on the basis of recognition of incidentally encoded face–profession pairs. The participants are asked to decide whether they had seen a given face–profession combination before or not and to rate the confidence of their decision on a three-point scale ranging from 1 = not sure to 3 = very sure. The accuracy of the corrected hit rates of the rearranged pairs was used here. 3. For the Scene Encoding Task [6], participants performed an incidental encoding task with 88 complex, gray-scaled images (44 indoor and 44 outdoor scenes; mean grey value 127, SD = 75) half of which are presented together with distractor images at the retrieval phase. The accuracy of an indoor/outdoor judgement (0-100%) of the corrected hit rates (hits minus false alarms) were used as scores here. 4. Finally, in the Object Location Task [7] sequences of 12 colored photographs of real-world objects are displayed at different locations in a 6-by-6 grid. After presentation, objects appear on the side of the screen and have to be moved to the correct locations by clicking on the objects and the locations with the computer mouse. The sum of all correct placed objects in two test trials

were used in the analyses. Overall, there were up to n=800 samples (buccal [n=678] and blood [n=800]) from BASE-II with test results for episodic memory performance available for DNAm profiling, of which 656 individuals had both buccal and blood samples available (**Table 1**; NB: buccal samples were processed in two separate laboratory batches: buccal-1 and buccal-2). The BASE-II/GendAge studies were conducted in accordance with the Declaration of Helsinki and approved by the ethics committee of the Charité – Universitätsmedizin Berlin (approval numbers: EA2/144/16, EA2/029/09) and the Max Planck Institute for Human Development, Berlin (approval numbers: LIP-2012-04). All participants gave written informed consent before participating.

Barcelona Brain Health Initiative (BBHI): The Barcelona Brain Health Initiative (BBHI) is an ongoing, longitudinal study with the focus on evaluating factors determining brain health [8]. EM performance was evaluated with the Face-Name Associative Memory Exam (S-FNAME, [9]), which includes scores of cued and delayed learning of names and occupations. Further, the Rey Auditory Verbal Learning Test (RAVLT, [10]) was also taken into account to compute a score of EM and included total learning and delayed recall of words.
Overall, there were 341 samples (buccal) from BBHI with test results for episodic memory performance available for DNAm profiling (**Table 1**). The BBHI project was conducted in accordance with the Declaration of Helsinki and following the recommendations of the “Unió Catalana d’Hospitals” with written informed consent from all subjects. The protocol was approved by the *Unió Catalana d’Hospitals* (approval number: CEIC 17/06).

Episodic memory phenotypes

Prior to processing, samples were excluded from the analysis if they represented outliers with 4 standard deviations (SDs) from the mean for any of the individual test performance results, or the final phenotype variable. For the cross-sectional EM phenotype, the first principal component (PC) of memory test performances (see above) was calculated with a principal component analysis (PCA) using the *PCA* function in the R package FactoMineR [11]. This variable (PC1) was corrected for age at time of the assessment used for the cross-sectional phenotype using a linear regression model performed with the *lm* function in R. The residuals of this regression were used as outcome phenotypes in the cross-sectional EM EWAS analyses. If episodic memory performance test results were available for more than one timepoint, the timepoint closest to the sampling of DNA sampling was chosen for the cross-sectional analysis.

For the longitudinal change in the EM phenotype, the annual percentage change (*APC*) was calculated for each memory test separately using the following formula (2):

$$(1) \qquad APC = \frac{Var_{last} - Var_{first}}{interval x \frac{Var_{first} + Var_{last}}{2}} * 100$$

Var_{first} represents the test performance for the memory test at the first time point, Var_{last} represents the test performance for the memory test at the last time point, and *interval* is the time interval (in years) between first and last time point.

From these *APC* variables, the first PC was calculated and corrected for age at baseline, as described for the cross-sectional EM performance phenotype above. Overall, we observed a decrease in the test performance over the time interval of on average six years between baseline measurement and follow-up in the BASE-II dataset (**Supplementary Figure 1**).

DNA extraction and processing

Blood: Genomic DNA was extracted from EDTA whole blood samples at the LGC facility in Berlin, Germany, using the LGC "Plus XL manual kit", LGC, United Kingdom. Samples were subsequently stored at −20°C.

Buccal: DNA extraction from the buccal swabs was performed in the LIGA laboratory using the Buccal-Prep Plus DNA Isolation Kit (Isohelix, UK). All steps in the extraction procedure were conducted according to manufacturer's instructions. To assess concentration and purity of obtained DNA, we used a NanoDrop ONE spectrophotometer (Thermo Fisher Scientific, USA).

DNA methylation profiling

DNAm profiling was performed at IKMB at UKSH campus Kiel using the "Infinium MethylationEPIC" array (Illumina, Inc.), as described previously [12]. In brief, DNAm profiling was performed on aliquots of DNA extracts diluted to ~50 ng/µl concentration. Bisulfite conversion of DNA samples was performed using the EZ DNA Methylation kit (Zymo Research), following the alternative incubation conditions for the Illumina Infinium MethylationEPIC Array from the supplier. After hybridization to the EPIC array, scanning was performed on an iScan instrument (Illumina, Inc.) according to the manufacturer's instructions (Document#1000000077299v0). To minimize the potential for batch effects, DNA samples were processed in consecutive laboratory experiments. After calling the raw DNAm intensities with the iScan control software (v2.3.0.0; Illumina, Inc.), they were exported in idat format for downstream processing and analysis.

DNA methylation data processing and quality control

DNAm data processing and quality control (QC) was performed using the same procedures as described previously [12] unless noted otherwise. This entailed using the R (v. 3.6.1) package bigmelon with default settings, unless otherwise noted [13]. Idat files were loaded into R and β-values were calculated according to the following formula, with Imet being the intensity of the methylated signal, and Iume being the intensity of the unmethylated signal.

(1) $\beta = \text{Imet} / (\text{Imet} + \text{Iume} + 100)$

Samples were excluded from the analysis if (a) the bisulfite conversion efficiency was below 80% according to the *bscon* function in the bigmelon package, (b) the sample had a beadcount < 3 in more than 5% of all probes, (c) the sample had a detection p-value below 0.05 in more than 1% of all probes, (d) the sample was identified as an outlier according to the *outlyx* function in the bigmelon package using a threshold of 0.15, (e) the sample showed a large change in β-values after normalization according to the *qual* function in the bigmelon package with a threshold of 0.1, (f) the sample showed a discrepancy between predicted sex according to the Horvath multi-tissue epigenetic age predictor [14] and reported sex, or (g) there was a greater than 70% discrepancy between genotypes of 42 SNPs determined concurrently from the EPIC and GSA SNP genotyping array (see below). All samples were normalized with the *dasen* function of bigmelon.

Cell-type composition estimates were calculated with the R-package EpiDISH [15], followed by correction of the DNAm values for cell-type composition with the *removeBatchEffect* function in the R package limma [16]. For all statistical analyses, the DNAm β-values were used. For the BASE-II blood samples, cell-type composition data was also available, as measured using laboratory procedures (i.e. for eosinophiles, lymphocytes, monocytes, and neutrophiles). In general, these molecular data correlated very highly with the EpiDISH estimates (r coefficients ranging from 0.46 to 0.96, **Supplementary Figure 4**), underscoring the validity of the *in silico* cell-type composition procedure used here.

Epigenome-wide association analyses to identify differentially methylated probes

Statistical analyses to identify differentially methylated probes (DMPs) were performed in each dataset separately using linear regression models performed by the *lm* function in R and

the EM phenotype (residuals of the first PC regressed on age, see above) as a continuous outcome variable:

(2) EM ~ DNAm + sex + DNAm PCs + genetic PCs

To account for differences in the DNAm profiles due to technical (e.g. laboratory batch, microarray) and other factors we performed a PCA on a subset of uncorrelated CpGs in the cell-type corrected data as described previously [12] and included these DNAm PCs as covariates in the regression model. To account for differences in genetic ancestry we performed a PCA (using PLINK v1.9 "--pca") on an LD-pruned set of SNP markers (--indep-pairwise 1""500 150 0.2) derived from genome-wide SNP genotyping data generated in parallel on the same DNA samples in each individual using the Global Screening Array (GSA; Illumina, Inc) and included the resulting genetic PCs as covariates in the regression model. More details on the general genotyping and QC procedures can be found in ref [17]. The number of DNAm and genetic PCs included in the EWAS analyses was estimated using scree plots and differed slightly per dataset; more details can be found in **Table 1**. To increase power, sample-specific EWAS results were meta-analysed across all available buccal datasets utilizing a fixed-effect inverse-variance weighting approach using the function *metagen* in the R package "meta" [18]. Annotation of CpGs to specific gene regions was based on the Illumina manifest (v1.0 B5) for the EPIC array and the GREAT annotation tool [19].

Calculation of poly-epigenetic scores (PES) for general cognitive abilities and AD

PES were calculated for each individual based on the test statistics from a published blood-based EWAS on cognitive abilities [20], on all phenotypes that were evaluated in that publication: general cognitive ability (g), general fluid cognitive ability (gf), vocabulary, digit symbol test score (digit), logical memory (LM), and verbal fluency (verbal). As the authors of ref. (30) did not provide *p*-values for their results, we calculated *p*-values using the effect sizes (β) and standard errors (SE) provided in the respective publication [20] to estimate phenotypic variance explained at different significance thresholds (i.e. $p<1 \times 10^{-4}$, $p<1 \times 10^{-5}$, $p<1 \times 10^{-6}$, $p<1 \times 10^{-7}$) we calculated *p*-values for the published test statistics [20] using the effect sizes (β) and standard errors (SE) with the following formula:

(3) $p = e^{\frac{-0.717*\beta}{SE} - 0.416*\left(\frac{\beta}{SE}\right)^2}$

For each *p*-value threshold, we created a set of uncorrelated CpGs by dividing the genome into 100kb bins followed by the selection of the CpG with the most extreme effect size per bin, a procedure analogous to SNP clumping for polygenic score (PGS) estimation in GWAS

[21]. Then, the effect sizes of the previous study [20] (β) and the normalized, scaled DNAm values (*CpG*) for each of the *n* uncorrelated CpGs for each *p*-value threshold were combined as follows:

(4) $\sum_{i=1}^{n} \beta_i CpG_i$

The same procedure was followed for AD-based EWAS results, here using test statistics from an EWAS on AD performed in entorhinal cortex (EC) brain samples recently completed by our group [12].

In addition, we performed linear regression analyses of cross-sectional and longitudinal change in EM on PES, adjusting for the same covariates as in the primary EWAS. All PES calculations were repeated using all available CpG-probes and not filtering for *p*-values. The two batches of BASE-II buccal samples were combined in one linear regression model, using the batch as a dummy variable.

Epigenetic age estimation

To estimate the "epigenetic age" (i.e. DNAm age) we applied the "Horvath multi-tissue predictor" (HMTP) using the R script provided in the primary publication [14]. This script utilizes the DNAm raw data after prior removal of probes failing QC. DNAm age acceleration was defined as the residual of a linear regression of epigenetic age on chronological age. This estimate of epigenetic age acceleration was then used as independent variable to predict EM performance using the same linear models as for the primary EWAS analyses, but also including cell-type composition estimates according to the R-package EpiDISH [15] as a covariate. Furthermore, these analyses used the EM performance measures without age adjustment, as age is already included in the HMTP algorithm.

Look-up of EM-associated CpGs in independent EWAS on AD-related phenotypes in human EC

To further characterize the CpGs with suggestive evidence of association with EM, we used test statistics from our recent AD EWAS of DNAm in EC [12]. Briefly, in that study, a meta-analysis of three different DNAm-based EWAS in EC of AD cases and controls for both AD Braak stage (n=320) and case-control status (n=337) was performed. The three EWAS datasets comprised newly generated EC DNAm data [12] and two previously published EC datasets (GEO accession numbers GSE59685 and GSE105109) [22,23]. Results for EM-associated CpGs identified in this study were obtained for the Braak stage and AD case-

control analyses generated in our previous study [12]. Additional association results were retrieved from EWAS on AD Braak stage [23–26] and cognition [20].

DNAm-mRNA correlation analyses

To estimate whether the DNAm patterns of CpGs showing suggestive association with EM in this study correlated with gene expression in human brain samples, we correlated DNAm status with RNA sequencing results generated in EC samples from healthy controls from ref. [12]. For more details on RNA sequencing procedures and data processing in these samples see [27]. Briefly, the analyses performed here entailed computing Spearman rank correlations using R's *cor.test* function between DNAm of a CpG and normalized RNA-seq data of the annotated gene(s). Multiple testing was accounted for by computing thresholds using the false-discovery rate (FDR) applying the Benjamini-Hochberg method. See ref. [12] for more details on data pre-processing and the Spearman rank correlations for CpG vs. mRNA levels.

Supplementary Figures

Supplementary Figure S1: Flowchart summarizing the workflow of this study.

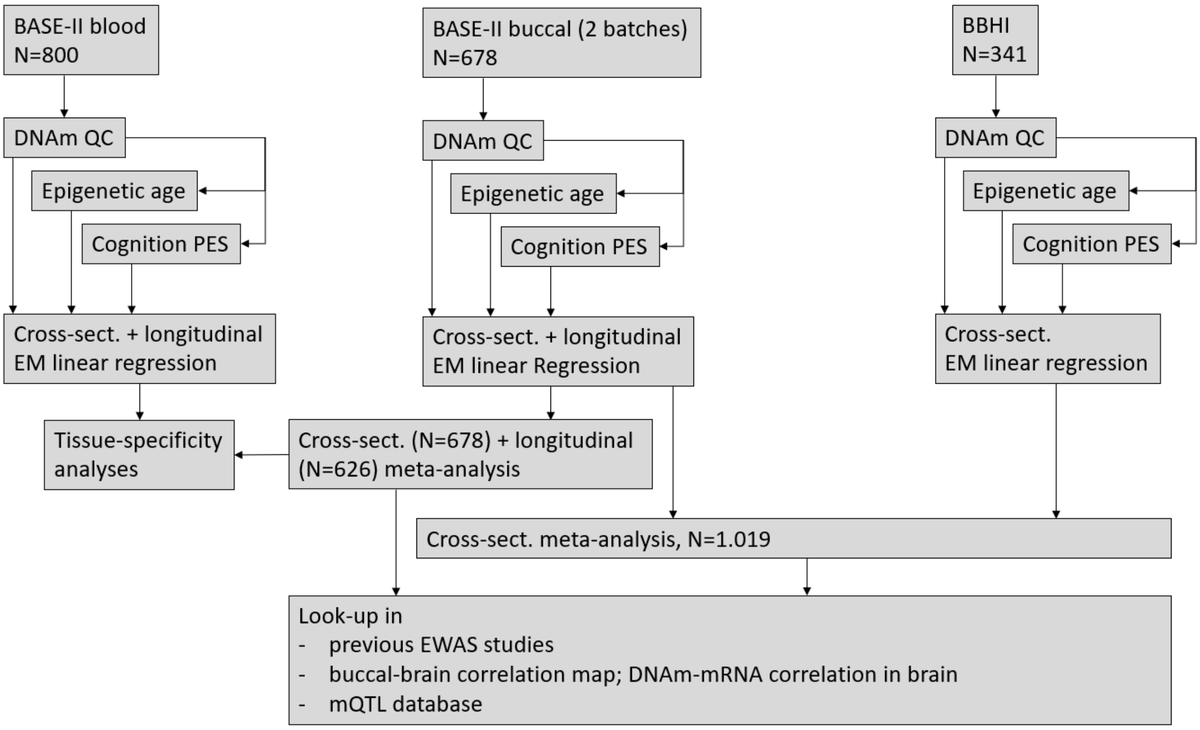

Supplementary Figure S1 legend: For the Berlin Aging Study II (BASE-II) datasets, both cross-sectional and longitudinal episodic memory performance (EM) data, and blood and buccal samples were available. The BASE-II buccal dataset was processed in two batches, which were subsequently meta-analysed (see Table 1 and Methods for more details). For the Barcelona Brain Health Initiative (BBHI) dataset, only the cross-sectional EM phenotype was available. In addition to the epigenome-wide as-sociation study (EWAS), the association of EM performance with Horvath epigenetic age and poly-epigenetic scores (PES) calculated according to summary statistics from McCartney et al. was estimated for each dataset.

Supplementary Figure S2: Trajectories of a random set of 100 individuals for test performance scores at TP0 and TP1 and boxplots of the test performance scores in all BASE-II individuals at baseline (TP0) and follow-up (TP1).

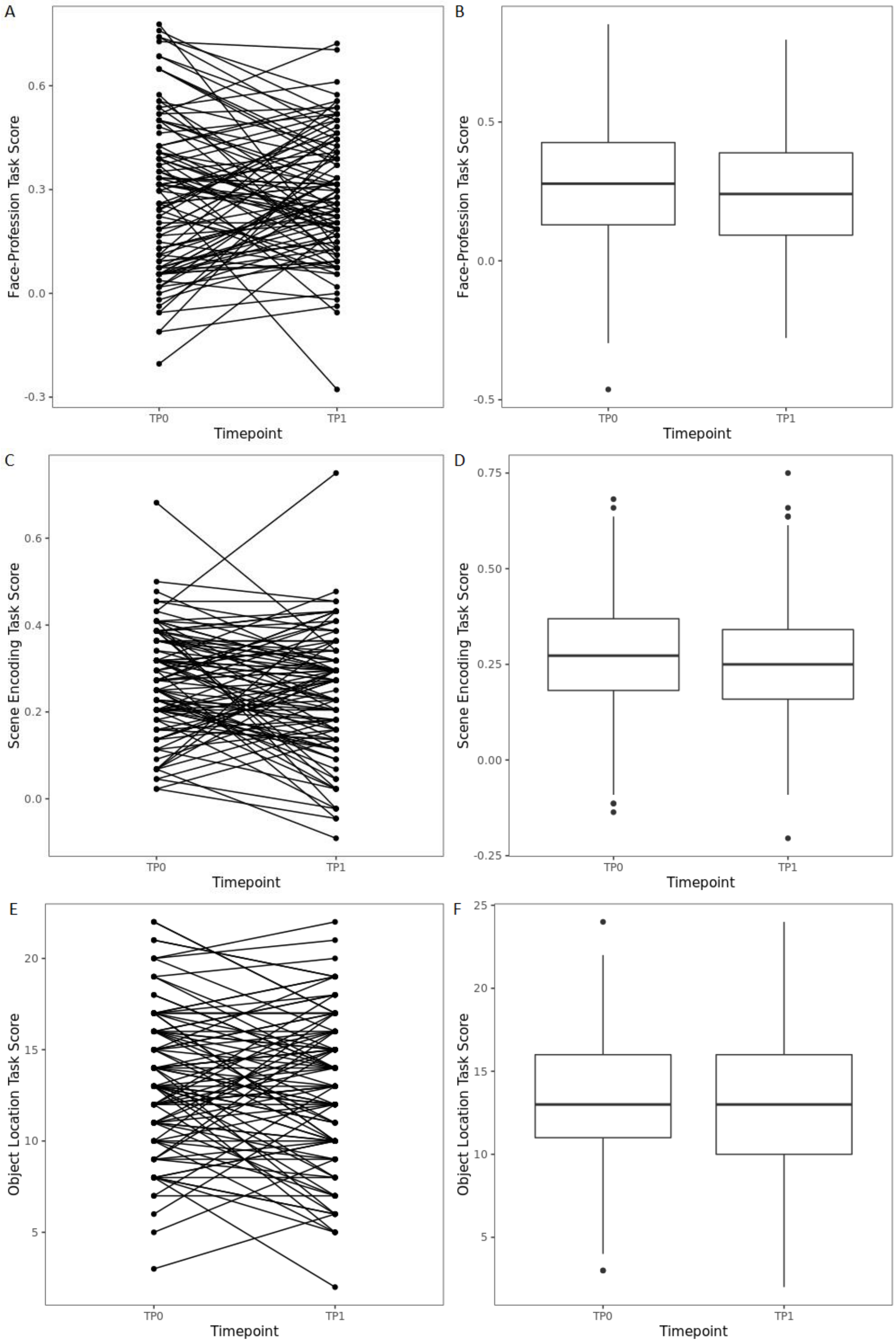

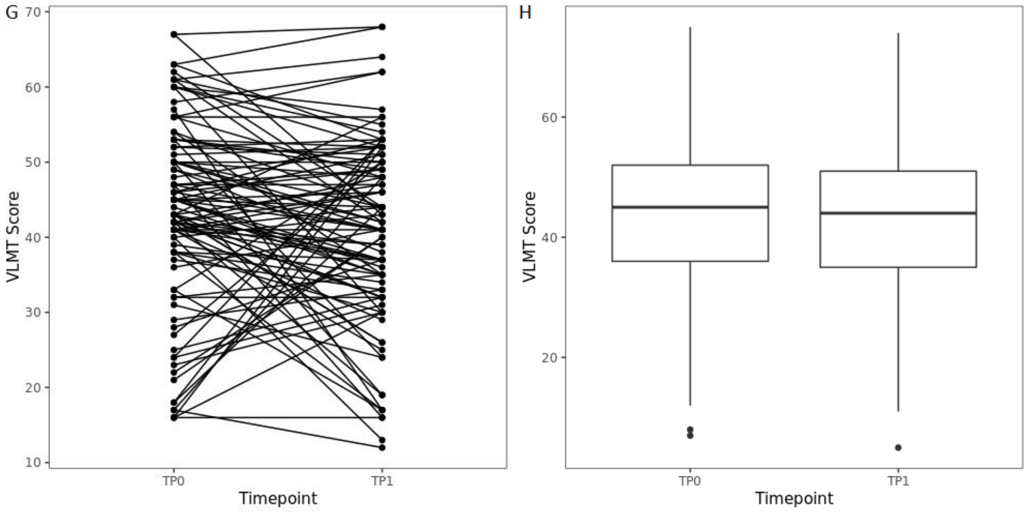

Supplementary Figure S2 Legend: Panels A-B: Face Profession Task; Panels C-D: Scene Encoding Task; Panels E-F: Object Location Task; Panels G-H: VLMT;
According to a paired t-test, the change of test scores between TP0 and TP1 was significantly different from 0 for the Face Profession Task (*p*=1.90E-03), the Scene Encoding Task (*p*=1.54E-08), and the Object Location Task (*p*=7.78E-06). The test performance for the VLMT did not differ significantly (*p*=0.40).

Supplementary Figure S3: Scatterplot of chronological age and HMTP DNAm age estimates for the BASE-II blood dataset (A, R=0.49) and the BASE-II buccal datasets (B, R=0.31) using individuals where both blood and buccal samples (n=656) were available.

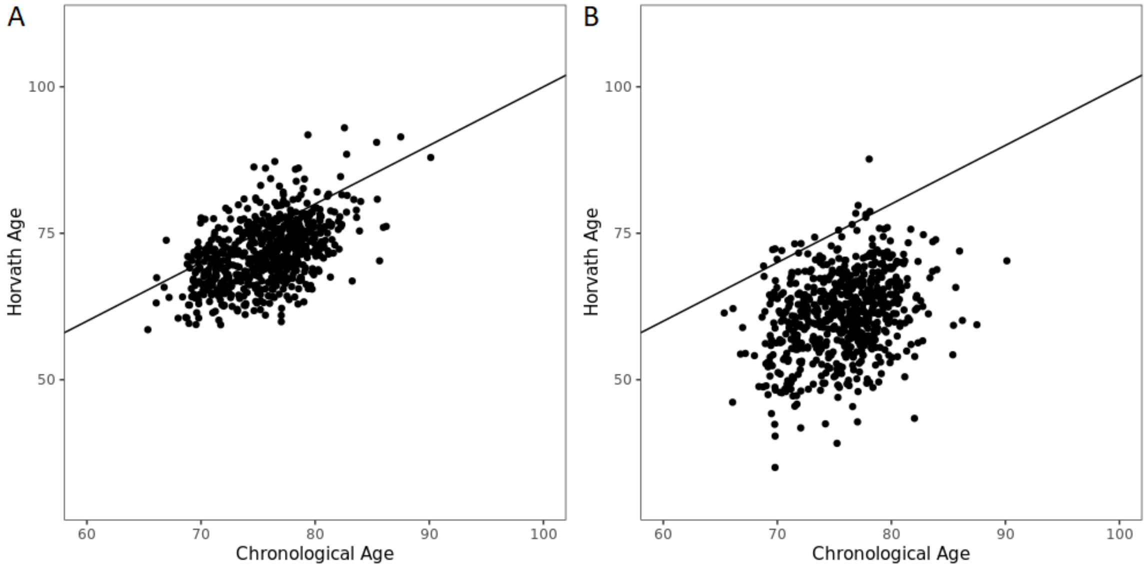

Supplementary Figure S3 Legend: Scatterplots of chronological age (x-axis) and HMTP DNAm age estimates (y-axis). The black line indicates perfect concordance between chronological and DNAm age; Panel A: BASE-II blood; Panel B: BASE-II buccal batch 1 and batch 2

Supplementary Figure S4: HMTP age acceleration and cross-sectional (left) and longitudinal change in (right) EM performance. A-B: BASE-II blood; C-D: BASE-II buccal-1; E-F: BASE-II buccal-2; G: BBHI.

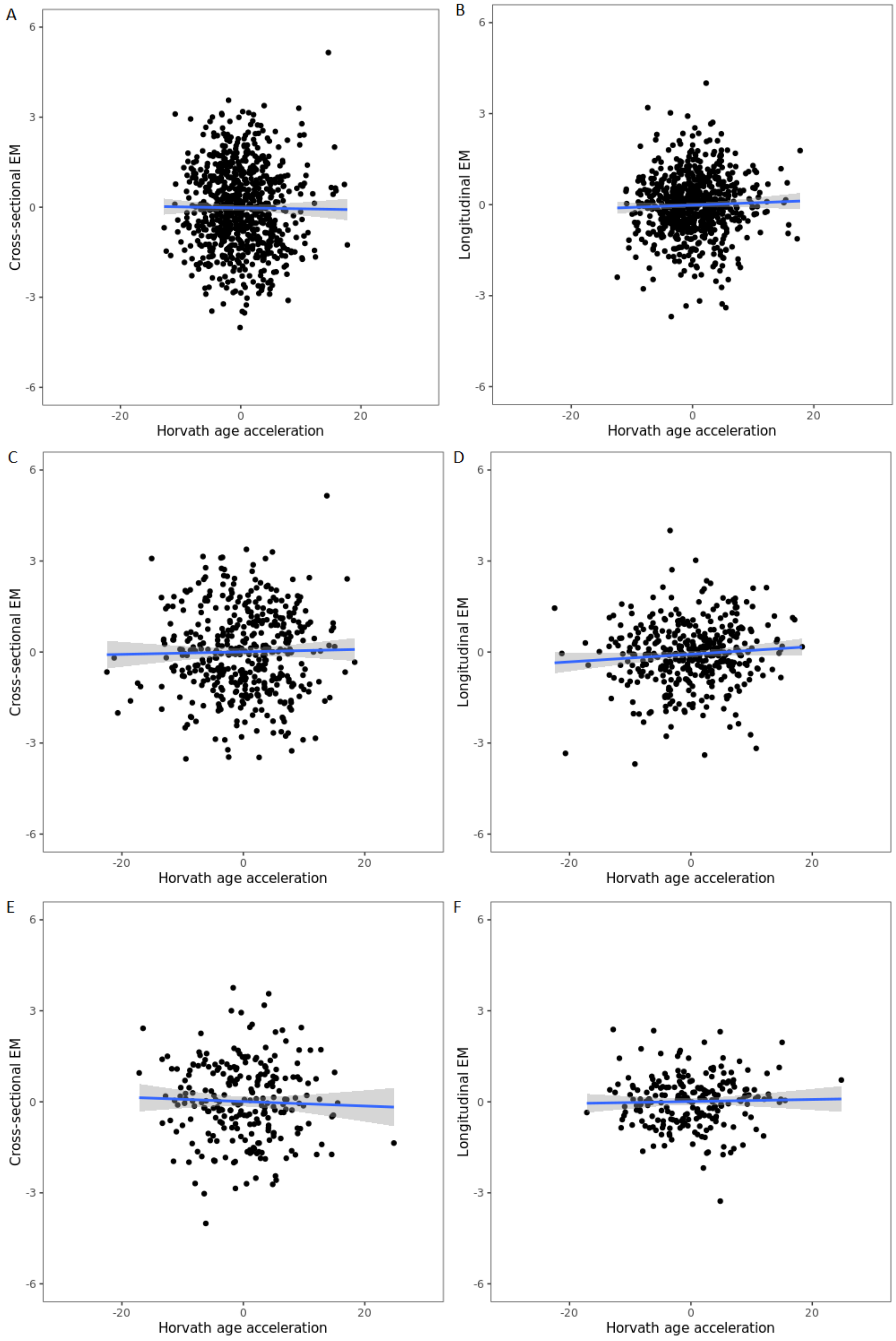

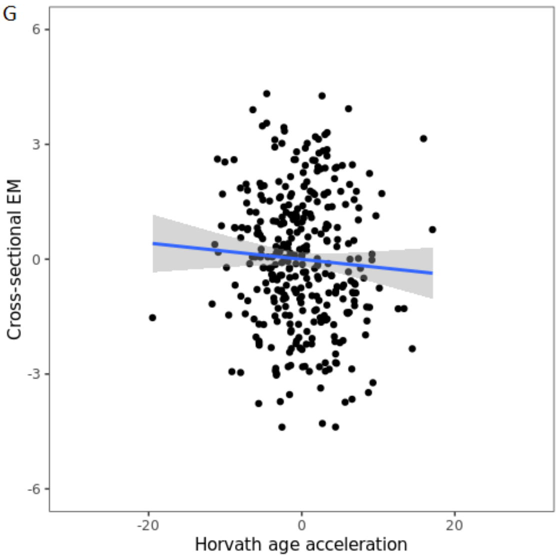
G
6
3
0
-3
-6
Cross-sectional EM
-20
0
20
Horvath age acceleration

Supplementary Figure S5: Comparison of cell-type compositions for eosinophiles (A), lymphocytes (B), monocytes (C), and neutrophiles (D) reported by a laboratory and estimated by the R-package EpiDISH [15] for the BASE-II blood samples

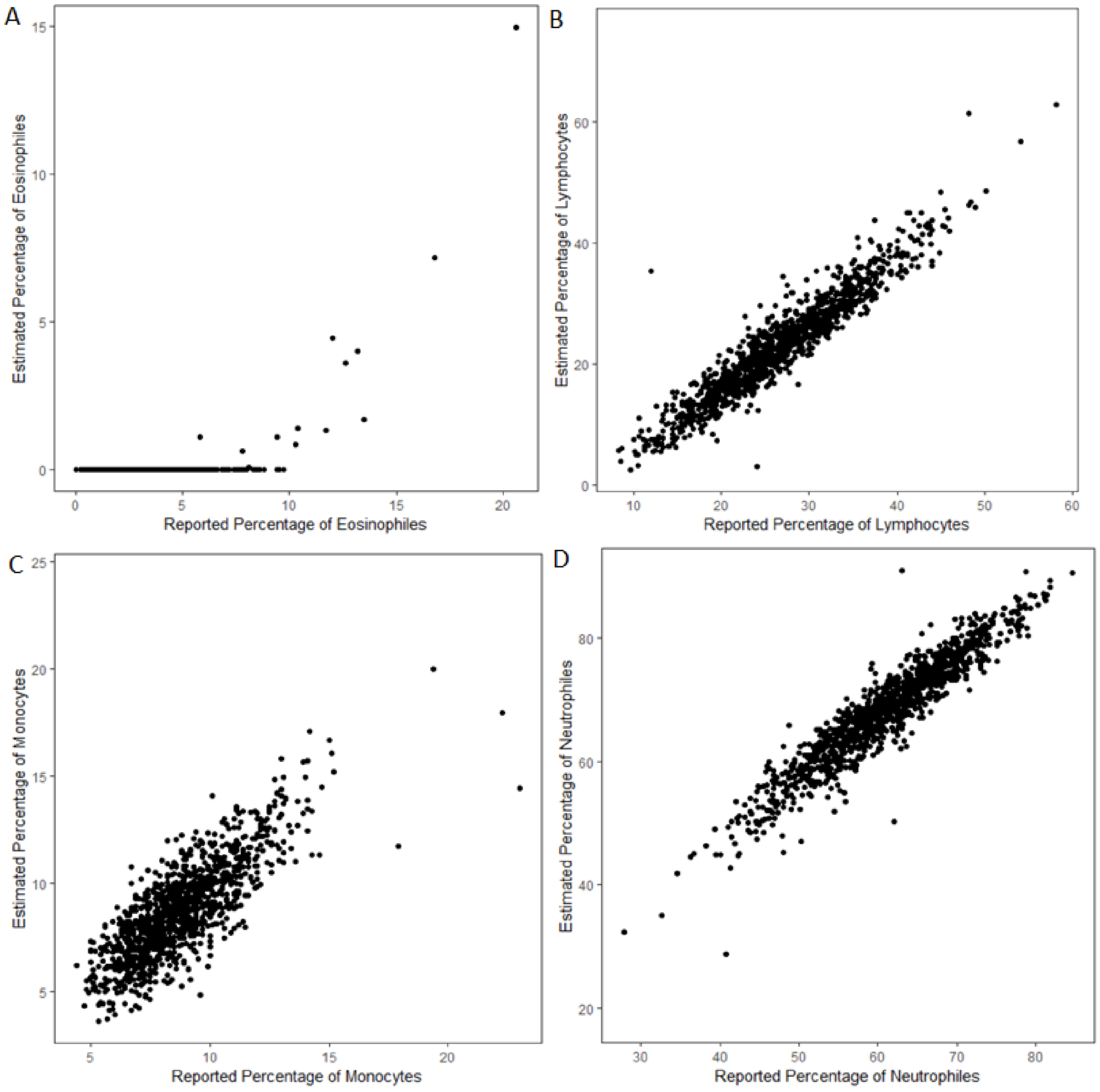

References

1. Bertram, L.; Böckenhoff, A.; Demuth, I.; Düzel, S.; Eckardt, R.; Li, S. C.; Lindenberger, U.; Pawelec, G.; Siedler, T.; Wagner, G. G.; Steinhagen-Thiessen, E. Cohort Profile: The Berlin Aging Study II (BASE-II). *Int. J. Epidemiol.* **2014**, *43* (3), 703–712. https://doi.org/10.1093/IJE/DYT018.
2. Gerstorf, D.; Bertram, L.; Lindenberger, U.; Pawelec, G.; Demuth, I.; Steinhagen-Thiessen, E.; Wagner, G. G. Editorial. *Gerontology* **2016**, *62* (3), 311–315. https://doi.org/10.1159/000441495.
3. Demuth, I.; Banszerus, V.; Drewelies, J.; Düzel, S.; Seeland, U.; Spira, D.; Tse, E.; Braun, J.; Steinhagen-Thiessen, E.; Bertram, L.; Thiel, A.; Lindenberger, U.; Regitz-Zagrosek, V.; Gerstorf, D. Cohort Profile: Follow-up of a Berlin Aging Study II (BASE-II) Subsample as Part of the GendAge Study. *BMJ Open* **2021**, *11* (6), e045576. https://doi.org/10.1136/BMJOPEN-2020-045576.
4. Helmstaedter, C.; Durwen, H. F. VLMT: Verbaler Lern- Und Merkfähigkeitstest: Ein Praktikables Und Differenziertes Instrumentarium Zur Prüfung Der Verbalen Gedächtnisleistungen. *Schweizer Arch. für Neurol. und Psychiatr.* **1990**, *141* (1), 21–30.
5. Schacter, D. L.; Osowiecki, D.; Kaszniak, A. W.; Kihlstrom, J. F.; Valdiserri, M. Source Memory: Extending the Boundaries of Age-Related Deficits. *Psychol. Aging* **1994**, *9* (1), 81–89. https://doi.org/10.1037/0882-7974.9.1.81.
6. Düzel, E.; Schütze, H.; Yonelinas, A. P.; Heinze, H.-J. Functional Phenotyping of Successful Aging in Long-Term Memory: Preserved Performance in the Absence of Neural Compensation. *Hippocampus* **2011**, *21* (8), 803–814. https://doi.org/10.1002/HIPO.20834.
7. Schmiedek, F.; Lövdén, M.; Lindenberger, U. Hundred Days of Cognitive Training Enhance Broad Cognitive Abilities in Adulthood: Findings from the COGITO Study. *Front. Aging Neurosci.* **2010**, *2* (JUL), 27. https://doi.org/10.3389/fnagi.2010.00027.
8. Cattaneo, G.; Bartrés-Faz, D.; Morris, T. P.; Sánchez, J. S.; Macià, D.; Tarrero, C.; Tormos, J. M.; Pascual-Leone, A. The Barcelona Brain Health Initiative: A Cohort Study to Define and Promote Determinants of Brain Health. *Front. Aging Neurosci.* **2018**, *10* (OCT), 321. https://doi.org/10.3389/fnagi.2018.00321.
9. Alviarez-Schulze, V.; Cattaneo, G.; Pachón-García, C.; Solana-Sánchez, J.; Tormos-Muñoz, J. M.; Alegret, M.; Pascual-Leone, A.; Bartrés-Faz, D. Validation and Normative Data of the Spanish Version of the Face Name Associative Memory Exam (S-FNAME). *J. Int. Neuropsychol. Soc.* **2022**, *28* (1), 74–84. https://doi.org/10.1017/S1355617721000084.
10. Rey, A. *L'examen Clinique En Psychologie.*, [1. éd.].; Presses universitaires de France: Paris, 1958.
11. Lê, S.; Josse, J.; Husson, F. FactoMineR: An R Package for Multivariate Analysis. *J. Stat. Softw.* **2008**, *25* (1), 1–18. https://doi.org/10.18637/JSS.V025.I01.
12. Sommerer, Y.; Dobricic, V.; Schilling, M.; Ohlei, O.; Sabet, S. S.; Wesse, T.; Fuß, J.; Franzenburg, S.; Franke, A.; Parkkinen, L.; Lill, C. M.; Bertram, L. Entorhinal Cortex EWAS Meta-Analysis Highlights Four Novel Loci Showing Differential Methylation in Alzheimer's Disease. *bioRxiv* **2021**, 2021.07.02.450878. https://doi.org/10.1101/2021.07.02.450878.
13. Gorrie-Stone, T. J.; Smart, M. C.; Saffari, A.; Malki, K.; Hannon, E.; Burrage, J.; Mill, J.; Kumari, M.; Schalkwyk, L. C. Bigmelon: Tools for Analysing Large DNA Methylation Datasets. *Bioinformatics* **2019**, *35* (6), 981–986. https://doi.org/10.1093/BIOINFORMATICS/BTY713.
14. Horvath, S. DNA Methylation Age of Human Tissues and Cell Types. *Genome Biol.* **2013**, *14* (10), 1–20. https://doi.org/10.1186/GB-2013-14-10-R115.

15. Teschendorff, A. E.; Breeze, C. E.; Zheng, S. C.; Beck, S. A Comparison of Reference-Based Algorithms for Correcting Cell-Type Heterogeneity in Epigenome-Wide Association Studies. *BMC Bioinformatics* **2017**, *18* (1), 1–14. https://doi.org/10.1186/S12859-017-1511-5.
16. Ritchie, M. E.; Phipson, B.; Wu, D.; Hu, Y.; Law, C. W.; Shi, W.; Smyth, G. K. Limma Powers Differential Expression Analyses for RNA-Sequencing and Microarray Studies. *Nucleic Acids Res.* **2015**, *43* (7), e47–e47. https://doi.org/10.1093/NAR/GKV007.
17. Hong, S.; Prokopenko, D.; Dobricic, V.; Kilpert, F.; Bos, I.; Vos, S. J. B.; Tijms, B. M.; Andreasson, U.; Blennow, K.; Vandenberghe, R.; Cleynen, I.; Gabel, S.; Schaeverbeke, J.; Scheltens, P.; Teunissen, C. E.; Niemantsverdriet, E.; Engelborghs, S.; Frisoni, G.; Blin, O.; Richardson, J. C.; Bordet, R.; Molinuevo, J. L.; Rami, L.; Kettunen, P.; Wallin, A.; Lleó, A.; Sala, I.; Popp, J.; Peyratout, G.; Martinez-Lage, P.; Tainta, M.; Dobson, R. J. B.; Legido-Quigley, C.; Sleegers, K.; Van Broeckhoven, C.; ten Kate, M.; Barkhof, F.; Zetterberg, H.; Lovestone, S.; Streffer, J.; Wittig, M.; Franke, A.; Tanzi, R. E.; Visser, P. J.; Bertram, L. Genome-Wide Association Study of Alzheimer's Disease CSF Biomarkers in the EMIF-AD Multimodal Biomarker Discovery Dataset. *Transl. Psychiatry* **2020**, *10* (1), 1–12. https://doi.org/10.1038/s41398-020-01074-z.
18. Balduzzi, S.; Rücker, G.; Schwarzer, G. How to Perform a Meta-Analysis with R: A Practical Tutorial. *Evid. Based. Ment. Health* **2019**, *22* (4), 153–160. https://doi.org/10.1136/EBMENTAL-2019-300117.
19. McLean, C. Y.; Bristor, D.; Hiller, M.; Clarke, S. L.; Schaar, B. T.; Lowe, C. B.; Wenger, A. M.; Bejerano, G. GREAT Improves Functional Interpretation of Cis-Regulatory Regions. *Nat. Biotechnol.* **2010**, *28* (5), 495–501. https://doi.org/10.1038/nbt.1630.
20. McCartney, D. L.; Hillary, R. F.; Conole, E. L. S.; Banos, D. T.; Gadd, D. A.; Walker, R. M.; Nangle, C.; Flaig, R.; Campbell, A.; Murray, A. D.; Maniega, S. M.; Valdés-Hernández, M. del C.; Harris, M. A.; Bastin, M. E.; Wardlaw, J. M.; Harris, S. E.; Porteous, D. J.; Tucker-Drob, E. M.; McIntosh, A. M.; Evans, K. L.; Deary, I. J.; Cox, S. R.; Robinson, M. R.; Marioni, R. E. Blood-Based Epigenome-Wide Analyses of Cognitive Abilities. *Genome Biol. 2022 231* **2022**, *23* (1), 1–16. https://doi.org/10.1186/S13059-021-02596-5.
21. Choi, S. W.; O'Reilly, P. F. PRSice-2: Polygenic Risk Score Software for Biobank-Scale Data. *Gigascience* **2019**, *8* (7), 1–6. https://doi.org/10.1093/GIGASCIENCE/GIZ082.
22. Smith, A. R.; Smith, R. G.; Pishva, E.; Hannon, E.; Roubroeks, J. A. Y.; Burrage, J.; Troakes, C.; Al-Sarraj, S.; Sloan, C.; Mill, J.; van den Hove, D. L.; Lunnon, K. Parallel Profiling of DNA Methylation and Hydroxymethylation Highlights Neuropathology-Associated Epigenetic Variation in Alzheimer's Disease. *Clin. Epigenetics* **2019**, *11* (1), 1–13. https://doi.org/10.1186/S13148-019-0636-Y.
23. Lunnon, K.; Smith, R.; Hannon, E.; De Jager, P. L.; Srivastava, G.; Volta, M.; Troakes, C.; Al-Sarraj, S.; Burrage, J.; Macdonald, R.; Condliffe, D.; Harries, L. W.; Katsel, P.; Haroutunian, V.; Kaminsky, Z.; Joachim, C.; Powell, J.; Lovestone, S.; Bennett, D. A.; Schalkwyk, L. C.; Mill, J. Methylomic Profiling Implicates Cortical Deregulation of ANK1 in Alzheimer's Disease. *Nat. Neurosci.* **2014**, *17* (9), 1164–1170. https://doi.org/10.1038/nn.3782.
24. Zhang, L.; Silva, T. C.; Young, J. I.; Gomez, L.; Schmidt, M. A.; Hamilton-Nelson, K. L.; Kunkle, B. W.; Chen, X.; Martin, E. R.; Wang, L. Epigenome-Wide Meta-Analysis of DNA Methylation Differences in Prefrontal Cortex Implicates the Immune Processes in Alzheimer's Disease. *Nat. Commun.* **2020**, *11* (1), 1–13.

https://doi.org/10.1038/s41467-020-19791-w.
25. Battram, T.; Yousefi, P.; Crawford, G.; Prince, C.; Babei, M. S.; Sharp, G.; Hatcher, C.; Vega-Salas, M. J.; Khodabakhsh, S.; Whitehurst, O.; Langdon, R.; Mahoney, L.; Elliott, H. R.; Mancano, G.; Lee, M.; Watkins, S. H.; Lay, A. C.; Hemani, G.; Gaunt, T. R.; Relton, C. L.; Staley, J. R.; Suderman, M. The EWAS Catalog: A Database of Epigenome-Wide Association Studies. *OSF Prepr.* **2021**, 4. https://doi.org/10.31219/OSF.IO/837WN.
26. Smith, R. G.; Pishva, E.; Shireby, G.; Smith, A. R.; Roubroeks, J. A. Y.; Hannon, E.; Wheildon, G.; Mastroeni, D.; Gasparoni, G.; Riemenschneider, M.; Giese, A.; Sharp, A. J.; Schalkwyk, L.; Haroutunian, V.; Viechtbauer, W.; van den Hove, D. L. A.; Weedon, M.; Brokaw, D.; Francis, P. T.; Thomas, A. J.; Love, S.; Morgan, K.; Walter, J.; Coleman, P. D.; Bennett, D. A.; De Jager, P. L.; Mill, J.; Lunnon, K. A Meta-Analysis of Epigenome-Wide Association Studies in Alzheimer’s Disease Highlights Novel Differentially Methylated Loci across Cortex. *Nat. Commun. 2021 121* **2021**, *12* (1), 1–13. https://doi.org/10.1038/s41467-021-23243-4.
27. Dobricic, V.; Schilling, M.; Schulz, J.; Zhu, L.-S.; Zhou, C.-W.; Fuß, J.; Franzenburg, S.; Zhu, L.-Q.; Parkkinen, L.; Lill, C. M.; Bertram, L. Differential MicroRNA Expression Analyses across Two Brain Regions in Alzheimer’s Disease. *Transl. Psychiatry* **2022**, *12* (1), 1–9. https://doi.org/10.1038/s41398-022-02108-4.
